# ZMYND8 preferentially binds phosphorylated EZH2 to promote a PRC2-dependent to -independent function switch in hypoxia-inducible factor–activated cancer

**DOI:** 10.1073/pnas.2019052118

**Published:** 2021-02-15

**Authors:** Bo Tang, Rui Sun, Dejie Wang, Haoyue Sheng, Ting Wei, Liguo Wang, Jun Zhang, Thai H. Ho, Lu Yang, Qiang Wei, Haojie Huang

**Affiliations:** ^a^Department of Urology, Institute of Urology, West China Hospital, Sichuan University, 610041 Chengdu, China;; ^b^Department of Biochemistry and Molecular Biology, Mayo Clinic College of Medicine and Science, Rochester, MN 55905;; ^c^Department of Urology, Fudan University Shanghai Cancer Center, Shanghai 200032, China;; ^d^Department of Oncology, Shanghai Medical College, Fudan University, Shanghai 200032, China;; ^e^Division of Biomedical Statistics and Informatics, Mayo Clinic College of Medicine and Science, Rochester, MN 55905;; ^f^Department of Laboratory Medicine and Pathology, Mayo Clinic College of Medicine and Science, Scottsdale, AZ 85259;; ^g^Division of Hematology and Oncology, Department of Internal Medicine, Mayo Clinic College of Medicine and Science, Phoenix, AZ 85054;; ^h^Department of Urology, Mayo Clinic College of Medicine and Science, Rochester, MN 55905;; ^i^Mayo Clinic Cancer Center, Mayo Clinic College of Medicine and Science, Rochester, MN 55905

**Keywords:** ZMYND8, EZH2, PRC2, hypoxia, cancer

## Abstract

Both gene repressor (Polycomb-dependent) and activator (Polycomb-independent) functions of the Polycomb protein enhancer of zeste homolog 2 (EZH2) are implicated in cancer progression. EZH2 protein can be phosphorylated at various residues, such as threonine 487 (T487), by CDK1 kinase, and such phosphorylation acts as a Polycomb repressive complex 2 (PRC2) suppression “code” to mediate the gene repressor-to-activator switch of EZH2 functions. Here we demonstrate that the histone reader protein ZMYND8 is overexpressed in human clear cell renal cell carcinoma (ccRCC). ZMYND8 binds to EZH2, and their interaction is largely enhanced by CDK1 phosphorylation of EZH2 at T487. ZMYND8 depletion not only enhances Polycomb-dependent function of EZH2 in hypoxia-exposed breast cancer cells or von Hippel–Lindau (VHL)-deficient ccRCC cells, but also suppresses the FOXM1 transcription program. We further show that ZMYND8 is required for EZH2–FOXM1 interaction and is important for FOXM1-dependent matrix metalloproteinase (*MMP*) gene expression and EZH2-mediated migration and invasion of VHL-deficient ccRCC cells. Our results identify a previously uncharacterized role of the chromatin reader ZMYND8 in recognizing the PRC2-inhibitory phosphorylation “code” essential for the Polycomb-dependent to -independent switch of EZH2 functions. They also reveal an oncogenic pathway driving cell migration and invasion in hypoxia-inducible factor–activated (hypoxia or VHL-deficient) cancer.

## Significance

The multiple-domain protein ZMYND8 is well known as a chromatin reader that recognizes methylated, acetylated, and/or mutated histones. Here we report that ZMYND8 is overexpressed in human ccRCC, and that it preferentially binds T487-phosphorylated EZH2. We show that in hypoxia-stimulated or von Hippel–Lindau–deficient cells, ZMYND8 binding of EZH2 is important for maintenance of EZH2 phosphorylation level, inhibition of PRC2 complex formation, and enhanced interaction between EZH2 and FOXM1. Our results reveal a reader function of ZMYND8 in recognizing a phosphorylation “code” on a nonhistone protein. Our findings also identify ZMYND8 binding as a facet of regulation and function of EZH2 phosphorylation in the Polycomb-dependent to -independent switch of EZH2/PRC2 activities.

Hypoxia plays important roles in cancer progression by inducing stabilization of hypoxia-inducible factors (HIFs) (1, 2). HIF proteins function as transcription factors to mediate the expression of hypoxia-inducible genes (3). There are three members in the HIF protein family—HIF1, HIF2, and HIF3—each of which consists of an O_2_-regulated α subunit and a constitutively expressed β subunit (4–6). Under normoxic conditions, HIFα proteins are hydroxylated by prolyl hydroxylases and hydroxylated HIFα proteins are ubiquitinated by von Hippel–Lindau (VHL) E3 ubiquitin ligase, thereby undergoing proteasome degradation (7). The *VHL* gene is frequently deleted or inactively mutated in more than 80% of patients with clear cell renal cell carcinoma (ccRCC) (8). HIFα proteins become stabilized in VHL-deficient cells even under normoxic conditions (2), thereby inducing expression of genes involved in angiogenesis, cell migration and invasion, cell survival, and stem cell maintenance (3).

The Polycomb protein EZH2 is the catalytic subunit of Polycomb repressive complex 2 (PRC2), which represses gene transcription by catalyzing histone H3 lysine 27 trimethylation (H3K27me3) (9–12). EZH2 is often overexpressed in many cancer types, including prostate, breast, and kidney cancer, and EZH2 overexpression is associated with poor patient outcomes (13–15). In addition to the gene repression activity, the gene activator function of EZH2 is also linked to its oncogenic roles (16–19). It has been shown that phosphorylation of EZH2 at serine 21 (S21) and threonine 487 (T487) by PKB/AKT and CDK1, respectively, results in the dissociation of EZH2 from the PRC2 complex (20, 21). Similarly, AMPK-mediated phosphorylation of EZH2 at T311 also disrupts EZH2 interaction with SUZ12 and abolishes its gene repression function (22). Most importantly, S21 phosphorylation of EZH2 not only switches off its gene repressor function, but also turns on its gene activator activity by enhancing EZH2 interaction with transcription factors such as androgen receptor, NFκB, and STAT3 (16, 17, 19), stressing the importance of the noncanonical gene activator function of EZH2 in oncogenesis (18, 23). While EZH2 phosphorylation is highly implicated in its role in gene activation and cancer progression, how EZH2 phosphorylation is leveraged by other signaling pathways to contribute to cancer progression has remained unexplored.

The bromodomain protein zinc finger MYND-type containing 8 (ZMYND8) is initially identified as an activated protein-kinase C–binding protein (also termed PRKCBP1 or RACK7) (24). ZMYND8 acts as a dual reader of histone marks H3K4me1 and H3K14ac to inhibit expression of metastasis-linked genes (25). ZMYND8 can recruit KDM5C histone demethylase to negatively regulate enhancer H3K4me3 level and restrict enhancer overactivation (26). Acting through the bromodomain, ZMYND8 also regulates the transcription-associated DNA damage repair by recognizing acetylated histones (27, 28). ZMYND8 can recognize mutated histone H3.3 (H3.3G34R) detected in pediatric glioblastomas (29); however, it is unclear whether ZMYND8 can also regulate tumor biology by recognizing posttranslational modifications of nonhistone proteins.

In this paper, we identify a molecular mechanism by which PRC2 activity is restricted by ZMYND8. We demonstrate that ZMYND8 interacts with T487-phosphorylated EZH2 and disrupts EZH2 binding with other PRC2 components in ccRCC cells. We also show that ZMYND8 augments EZH2 interaction with FOXM1 and activates the transcription of matrix metalloproteinase (*MMP*) genes, thereby promoting EZH2 gene activator function and ccRCC cell migration and invasion.

## Results

### ZMYND8 Negatively Regulates PRC2 Target Gene Expression in Breast Cancer Cells under Hypoxia.

**Fig. 1.**
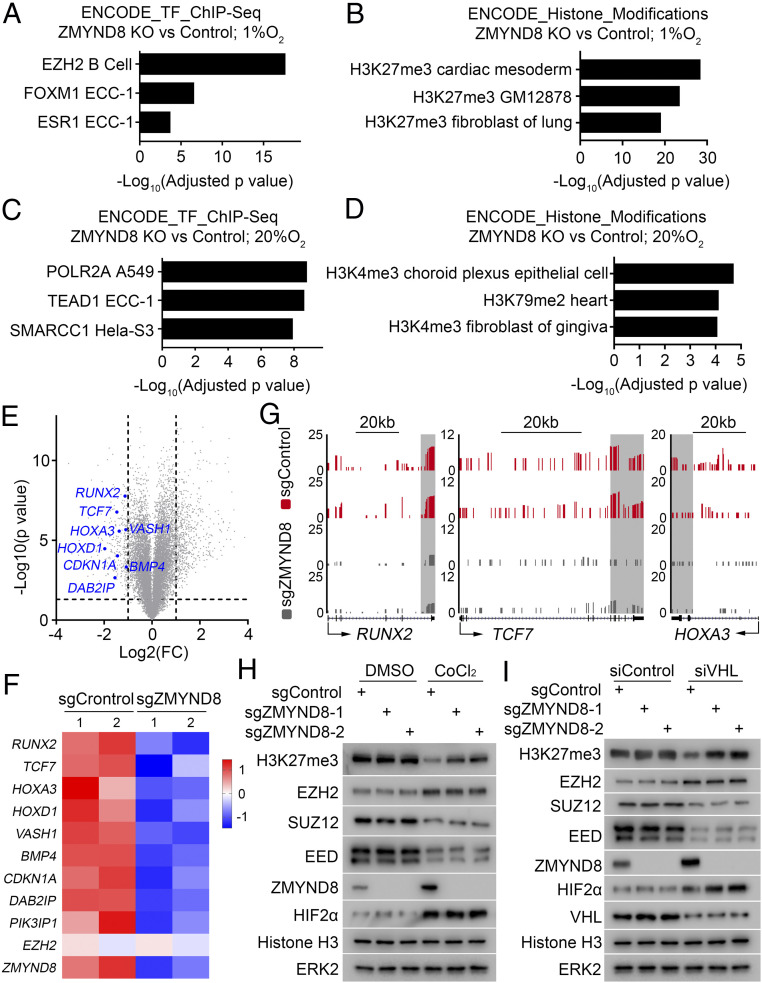
ZMYND8 negatively regulates PRC2 target gene expression in breast cancer cells under hypoxia. (*A*–*D*) Meta-analysis of down-regulated genes after ZMYND8 KO under hypoxia (*A* and *B*) or normoxia (*C* and *D*) by Enrichr (http://amp.pharm.mssm.edu/Enrichr). Data are ranked according to *P* value corrected by multiple hypothesis testing. Transcription factor (TF)-regulated (*A* and *C*) or histone modification-regulated (*B* and *D*) genes derived from the Encyclopedia of DNA Elements (ENCODE) dataset with the expression dysregulated genes in ZMYND8 KO cells were determined using Fisher’s exact test. (*E*) Volcano plot depicting the differentially expressed genes between ZMYND8 WT and KO MDA-MB-231 cells under hypoxia revealed by RNA-seq data. The names of the representative PRC2 target genes are indicated. (*F*) Heatmap showing expression of representative PRC2 target genes between ZMYND8 WT and KO MDA-MB-231 cells under hypoxia. (*G*) UCSC Genome Browser screen shot of RNA-seq tracks for well-known H3K27me3 target genes (*HOXA3*, *RUNX2*, and *TCF7*) commonly down-regulated by ZMYND8 KO under hypoxia. (*H*) WB analysis of the indicated proteins in lysate of control (sgControl) and ZMYND8 KO (sgZMYND8) MDA-MB-231 cells treated with or without the hypoxia mimetic reagent CoCl_2_ (200 µM) for 24 h before harvest. (*I*) Control (sgControl) and ZMYND8 KO (sgZMYND8) MDA-MB-231 cells were transfected with control siRNA (siControl) and VHL-specific siRNA (siVHL) for 48 h, and cells were harvested for WB analysis of the indicated proteins.

In an attempt to define novel functions of ZMYND8 in cancer, we analyzed a published RNA-seq dataset derived from ZMYND8 wild-type (WT) and knockout (KO) MDA-MB-231 breast cancer cells (30). Pathway analysis indicated that a subset of EZH2 and H3K27me3 target genes were down-regulated in ZMYND8 KO cells (Fig. 1 *A* and *B*). This phenomenon appears to be hypoxia-specific, since ZMYND8 KO failed to elicit similar results under normoxic conditions (Fig. 1 *C* and *D*). The top hits of ZMYND8 KO-affected H3K27me3 target genes are shown in volcano plots and a heatmap (Fig. 1 *E* and *F*). Analysis of RNA-seq data also showed that expression of canonical PRC2 target genes *HOXA3*, *RUNX2*, and *TCF7* (21) was down-regulated by ZMYND8 KO under hypoxia (Fig. 1*G*). We further confirmed that ZMYND8 KO increased the overall level of H3K27me3 in MDA-MB-231 breast cancer cells, and that this only occurred in cells under hypoxia (Fig. 1*H*).

The *VHL* gene is frequently deleted or mutated in ccRCC in patients and loss of VHL results in the stabilization of HIFα proteins, mimicking the role of hypoxia (8). As expected, similar to the effect of hypoxia (Fig. 1*H*), knockdown of VHL resulted in up-regulation of HIF2α protein in MDA-MB-231 cells under normoxic conditions (Fig. 1*I*). Similar to the results reported previously (18), we demonstrated that VHL knockdown or hypoxia exposure decreased the expression of PRC2 components SUZ12 and EED, consistent with the decreased level of H3K27me3 (Fig. 1 *H* and *I*). In contrast, EZH2 protein expression was substantially up-regulated under both conditions (Fig. 1 *H* and *I*). Surprisingly, we found that ZMYND8 KO increased the overall level of H3K27me3 in VHL-deficient cells but not in VHL-proficient cells under normoxic conditions (Fig. 1*I*), mimicking the scenario in MDA-MB-231 cells under hypoxia (Fig. 1*H*). Notably, ZMYND8 KO failed to affect expression of PRC2 proteins, including EZH2, SUZ12, and EED, regardless of hypoxia exposure or VHL deficiency (Fig. 1 *H* and *I*).

To determine whether HIFα proteins play a critical role in ZMYND8 regulation of H3K27me3 levels in ccRCC cells, we knocked down HIF1α and HIF2α in hypoxia-treated or *VHL*-deficient cells. HIF1/2α knockdown increased H3K27me3 and decreased PRC2 target gene expression but abolished the ZMYND8 knockdown-induced increase in H3K27me3 and decreased the expression of PRC2 target genes (*SI Appendix*, Fig. S1 *A*–*D*). Together, our findings show that ZMYND8 expression down-regulates H3K27me3 levels under HIFα activation conditions (hypoxia or VHL loss) without affecting expression of the key PRC2 components EZH2, SUZ12, and EED, implying the existence of a previously undefined role of ZMYND8 in regulating H3K27me3 and PRC2 activity.

### ZMYND8 Is Overexpressed and Associated with Poor Clinical Outcome in ccRCC Patients.

**Fig. 2.**
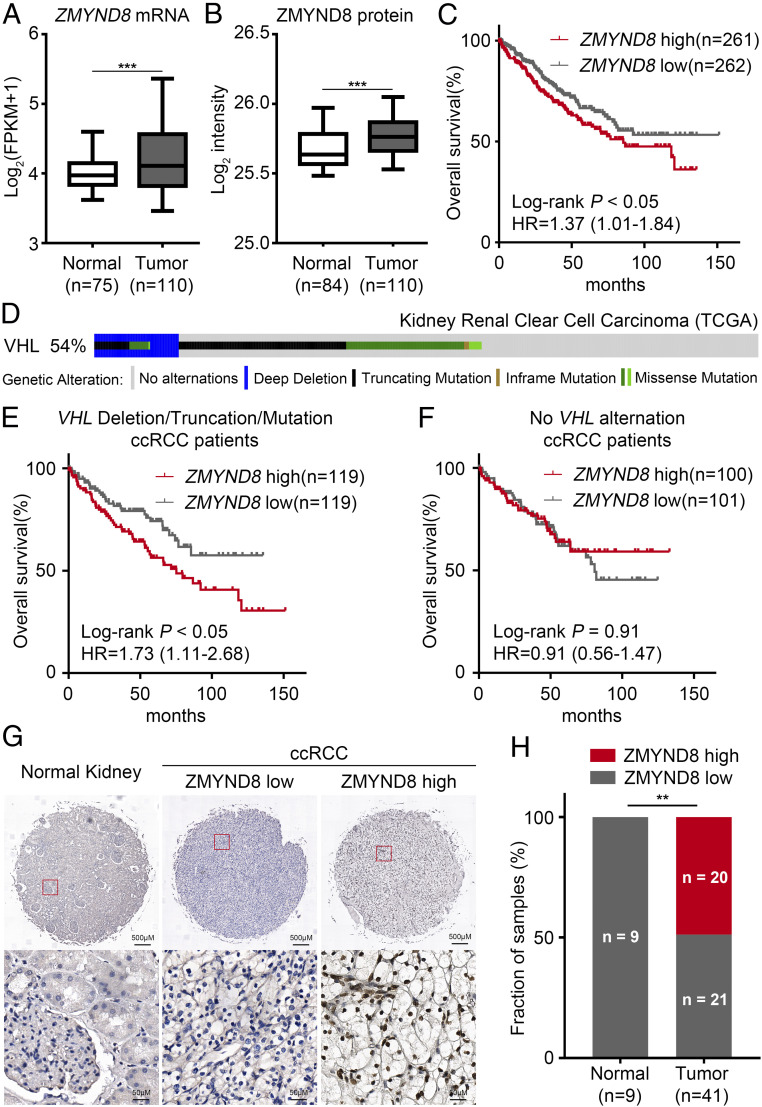
ZMYND8 is overexpressed in ccRCC and is associated with the poor clinical outcome in patients. (*A* and *B*) Analysis of ZMYND8 mRNA (*A*) and protein (*B*) levels in human ccRCC and normal kidney tissues from the CPTAC ccRCC dataset. ***P < 0.001. (*C*) Kaplan–Meier survival analysis for ccRCC patients from the TCGA dataset using the log-rank test. Patients were divided by median expression level of ZMYND8 mRNA. HR, hazard ratio. (*D*) Using cBioportal, OncoPrint images of genomic alternations of the *VHL* gene were generated for 448 ccRCC samples from the TCGA dataset for which *VHL* status was available. (*E* and *F*) Kaplan–Meier survival analysis using the log-rank test for TCGA ccRCC patients for whom *VHL* status and prognosis data were available. Results for patients with *VHL* deletion/truncation/mutation are shown in *E*, and results for patients without *VHL* alterations are shown in *F*. Patients were divided by median expression level of ZMYND8 mRNA. (*G*) Representative ZMYND8 IHC staining images from a human ccRCC and normal kidney TMA. (*H*) Quantification of ZMYND8 protein level in normal kidney tissues and ccRCC specimens. **P < 0.01, chi-square test.

Because ZMYND8 only regulates H3K27me3 levels under hypoxia or in VHL-deficient cells (Fig. 1 *H* and *I*), we chose to determine how ZMYND8 regulates PRC2 activities in ccRCC in which VHL is often deleted or mutated. Analysis of the Clinical Proteomic Tumor Analysis Consortium (CPTAC) ccRCC dataset (8) revealed that both ZMYND8 mRNA and protein expression are significantly elevated in primary ccRCC compared with adjacent normal kidney tissues (Fig. 2 *A* and *B*). Kaplan-Meier analysis of The Cancer Genome Atlas (TCGA) dataset revealed that high levels of ZMYND8 mRNA are associated with poor overall survival of ccRCC patients (Fig. 2*C*).

To explore whether the association of ZMYND8 overexpression with poor outcome of ccRCC patients is affected by the status of VHL gene alteration, we stratified the TCGA patients into two groups. We found that in the *VHL* deletion/truncation group (238 patients), high levels of ZMYND8 mRNA were associated with poor overall survival (Fig. 2 *D* and *E*). In contrast, there was no significant association of ZMYND8 mRNA expression with overall survival of patients lacking *VHL* alterations (201 patients) (Fig. 2 *D* and *F*). We further examined ZMYND8 expression in patient samples by performing immunohistochemistry (IHC) on a tissue microarray (TMA) containing a cohort of ccRCC specimens (41 ccRCC and 9 normal kidney TMA elements). Representative IHC images displaying high and low staining of ZMYND8 are shown in Fig. 2*G*. In this cohort, 20 of 41 (48.7%) TMA ccRCC specimens were ZMYND8-positive, but none of the normal kidney specimens expressed high levels of ZMYND8 (Fig. 2*H*). Together, these data indicate that ZMYND8 is overexpressed in a subset of ccRCC, and that its overexpression is associated with poor clinical outcome in ccRCC patients with *VHL* deficiency.

### ZMYND8 Negatively Regulates H3K27me3 Target Gene Expression in ccRCC Cells in Culture and in Patients.

**Fig. 3.**
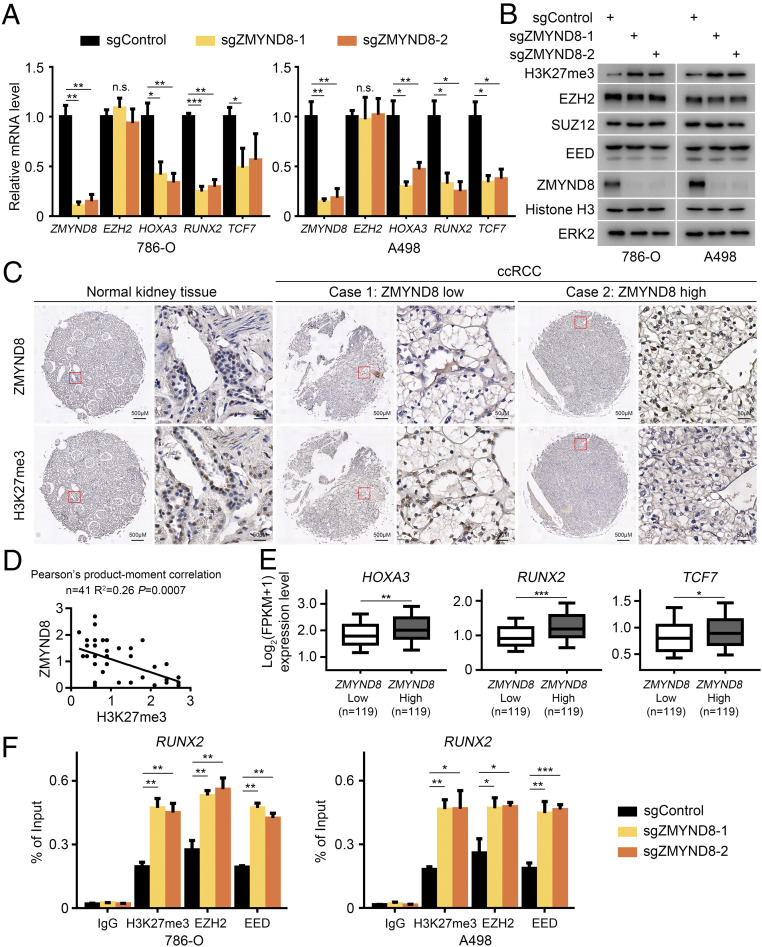
ZMYND8 negatively regulates H3K27me3 target gene expression in ccRCC cells in culture and in patients. (*A*) 786-O and A498 cells transfected with control (sgControl) or two independent ZMYND8-specific sgRNAs (sgZMYND8-1 and sgZMYND8-2) were harvested for RT-qPCR analysis of expression of the indicated genes. *P < 0.05; **P < 0.01; ***P < 0.001; n.s., not significant. (*B*) WB analysis of the indicated proteins in lysate of 786-O and A498 cells transfected with sgControl or sgZMYND8. (*C*) Representative images of IHC with anti-ZMYND8 and anti-H3K27me3 antibodies on TMA (9 normal kidney and 41 ccRCC TMA elements) tissue sections. (*D*) Correlation analysis of IHC staining of ZMYND8 and H3K27me3 proteins in ccRCC patient specimens. (*E*) Meta-analysis of mRNA expression of indicated PRC2 target genes in ZMYND8 high and low TCGA patient samples. **P < 0.01; ***P < 0.001. (*F*) 786-O and A498 sgControl or sgZMYND8 cells were harvested for ChIP-qPCR analysis of the occupancy of indicated proteins at the *RUNX2* gene locus. *P < 0.05; **P < 0.01; ***P < 0.001.

Next, we sought to explore the functional relevance of ZMYND8 regulation of PRC2 activity in ccRCC. To mimic the composition of PRC2 under hypoxic conditions, we performed our studies in two VHL-null ccRCC cell lines, 786-O and A498, to determine whether ZMYND8 affects EZH2 function and H3K27me3 level. We found that ZMYND8 knockout had no effect on EZH2 mRNA and protein expression (Fig. 3 *A* and *B*). ZMYND8 depletion also failed to affect protein expression of other PRC2 components examined, such as SUZ12 and EED (Fig. 3*B*). While ZMYND8 depletion had little or no effect on the expression of total histone H3 protein, it largely increased H3K27me3 levels in both 786-O and A498 cells (Fig. 3*B*), consistent with observation in MDA-MB-231 cells under hypoxic or VHL-depleted conditions (Fig. 1 *H* and *I*). The differential expression of several PRC2 targets genes (e.g., *HOXA3*, *RUNX2*, *TCF7*) in control and ZMYND8-deficient 786-O and A498 cells was further verified by RT-qPCR (Fig. 3*A*). ZMYND8 overexpression in *VHL*-null ccRCC cell lines with relatively low expression of endogenous ZMYND8 suppressed H3K27me3 levels without affecting EZH2, SUZ12, or EED protein expression (*SI Appendix*, Fig. S2 *A* and *B*).

We further examined ZMYND8 and H3K27me3 levels in a cohort of 9 normal kidney tissues and 41 ccRCC specimens. IHC staining of patient samples on TMA was evaluated by measuring both staining intensity and percentage of positive cells. Representative IHC images displaying high and low staining of ZMYND8 and H3K27me3 are shown in Fig. 3*C*. Further analysis showed that ZMYND8 protein expression was inversely correlated with H3K27me3 level in this cohort (Pearson’s product-moment correlation, *R*^2^ = 0.26; *P* = 0.0007) (Fig. 3*D*). We also analyzed the TCGA RNA-seq data from 238 *VHL-*deficient ccRCC patients and found that samples with higher ZMYND8 mRNA levels expressed higher levels of PRC2 target genes *HOXA3*, *RUNX2*, and *TCF7* (Fig. 3*E*).

To further understand the mechanism by which ZMYND depletion increases H3K27me3 level, we examined binding of the PRC2 complex in these gene promoters. Consistent with the finding that ZMYND depletion increased H3K27me3 levels in the promoter of PRC2 target gene *RUNX2*, it also increased occupancy of EZH2 and EED in the *RUNX2* promoter in both cell lines (Fig. 3*F*), although ZMYND8 depletion did not affect EZH2 and EED protein expression (Fig. 3*B*). These data indicate that ZMYND8 negatively regulates PRC2 occupancy at its target gene loci without affecting the expression of PRC2 components examined.

### ZMYND8 Interacts with EZH2 in ccRCC Cells.

**Fig. 4.**
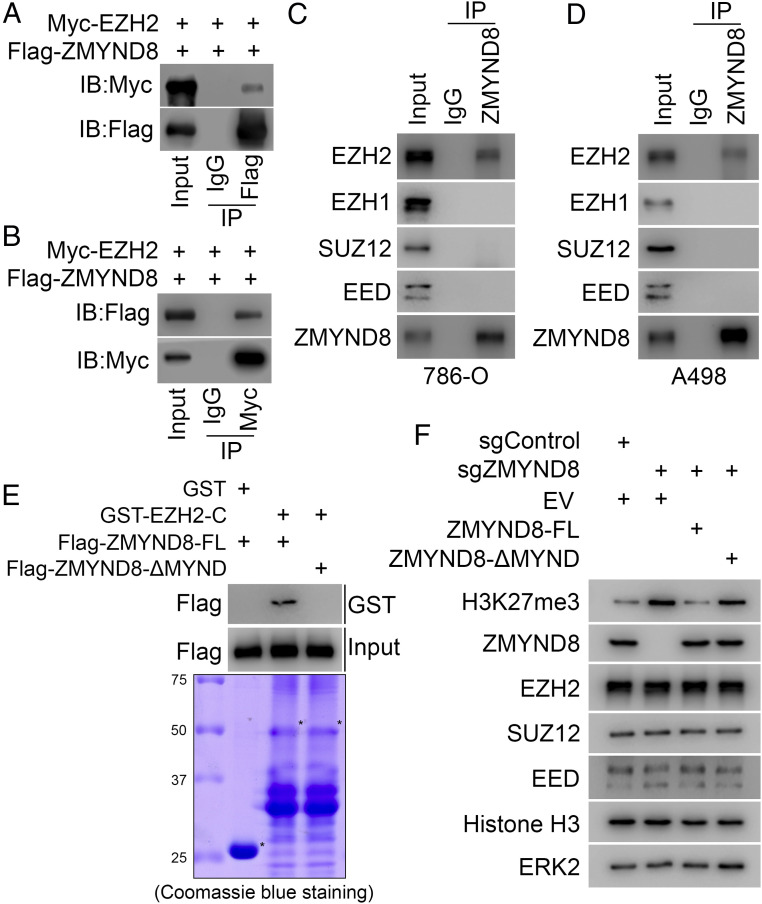
ZMYND8 interacts with EZH2 in ccRCC cells. (*A* and *B*) WB analysis of co-IP of ectopically expressed Flag-ZMYND8 and Myc-EZH2 in 293T cells. (*C* and *D*) WB analysis of co-IP of endogenous ZMYND8 and PRC2 components EZH2, SUZ12, and EED in 786-O cells (*C*) and A498 cells (*D*). (*E*) WB analysis of ZMYND8-FL and ZMYND8-ΔMYND proteins produced by the TNT Quick Coupled Transcript/Translation System pulled down by GST or GST-EZH2-C recombinant proteins. (*F*) A498 cells stably infected with lentivirus for sgControl or sgZMYND8 were transfected with empty vector (EV), ZMYND8-FL, or ZMYND8-ΔMYND. Cells were harvested for WB analysis of indicated proteins.

To further explore how ZMYND8 regulates PRC2 function and H3K27me3 level in ccRCC cells, we first examined whether ZMYND8 physically interacts with EZH2, a subunit of the PRC2 complex catalyzing H3K27me3 (31). We performed coimmunoprecipitation (co-IP) assays and demonstrated that ectopically expressed Myc-EZH2 protein was coimmunoprecipitated by Flag-ZMYND8 in 293T cells (Fig. 4*A*). Reciprocally, we found that Flag-ZMYND8 was coimmunoprecipitated by Myc-tagged EZH2 (Fig. 4*B*). Co-IP analysis further showed that ZMYND8 interacts with EZH2 at endogenous levels in two ccRCC cell lines examined, but no ZMYND8 binding of EZH1, SUZ12, or EED was detected (Fig. 4 *C* and *D*).

Next, we sought to identify the domain(s) involved in the interaction between ZMYND8 and EZH2. ZMYND8 contains multiple functional domains, including PHD finger, BROMO, and PWWP (for histone modification and DNA binding) and MYND at the C-terminal (involving protein–protein interactions) (27, 32). We first generated one N-terminal (ΔPBP) and three C-terminal (ΔC1, ΔC2 and ΔC3) truncation mutants from Flag-tagged ZMYND8 (*SI Appendix*, Fig. S3*A*). Full-length (FL) Flag-ZMYND8 and these truncation mutants were transfected into 293T cells for co-IP assays. We demonstrated that FL Flag-ZMYND8, ΔPBP, or ΔC3 deletion mutants interacted with EZH2. In contrast, ΔC1 or ΔC2 mutant lacking the MYND domain failed to interact with EZH2 (*SI Appendix*, Fig. S3*B*), indicating that the MYND domain is required for ZMYND8 interaction with EZH2.

To determine which fragment of EZH2 is involved in ZMYND8 binding, we purified four glutathione S-transferase (GST) EZH2 recombinant proteins from bacteria (*SI Appendix*, Fig. S3 *C* and *D*). GST pulldown assays showed that GST-EZH2-C (336-554), but not the other three truncation mutants of EZH2 or GST alone, specifically interacted with ZMYND8 in 786-O cell lysate (*SI Appendix*, Fig. S3*D*). Furthermore, we generated a Flag-tagged MYND domain deletion mutant of ZMYND8 and produced Flag-ZMYND8-FL and Flag-ZMYND8-ΔMYND proteins using the TNT Quick Coupled Transcript/Translation System (from Promega). In vitro protein binding assays showed that GST-EZH2-C specifically interacted with Flag-ZMYND8-FL rather than the MYND domain deletion mutant (Fig. 4*E*). Furthermore, we demonstrated that rescued expression of ZMYND8-WT, but not of ZMYND8-ΔMYND mutant, in ZMYND8-depleted A498 cells decreased H3K27me3 levels (Fig. 4*F*). Together, these data suggest that ZMYND8 directly binds to EZH2, and that ZMYND8 binding of EZH2 and inhibition of H3K27me3 is mediated through the MYND domain.

### T487 Phosphorylation by CDK1 Largely Enhances EZH2 Interaction with ZMYND8.

**Fig. 5.**
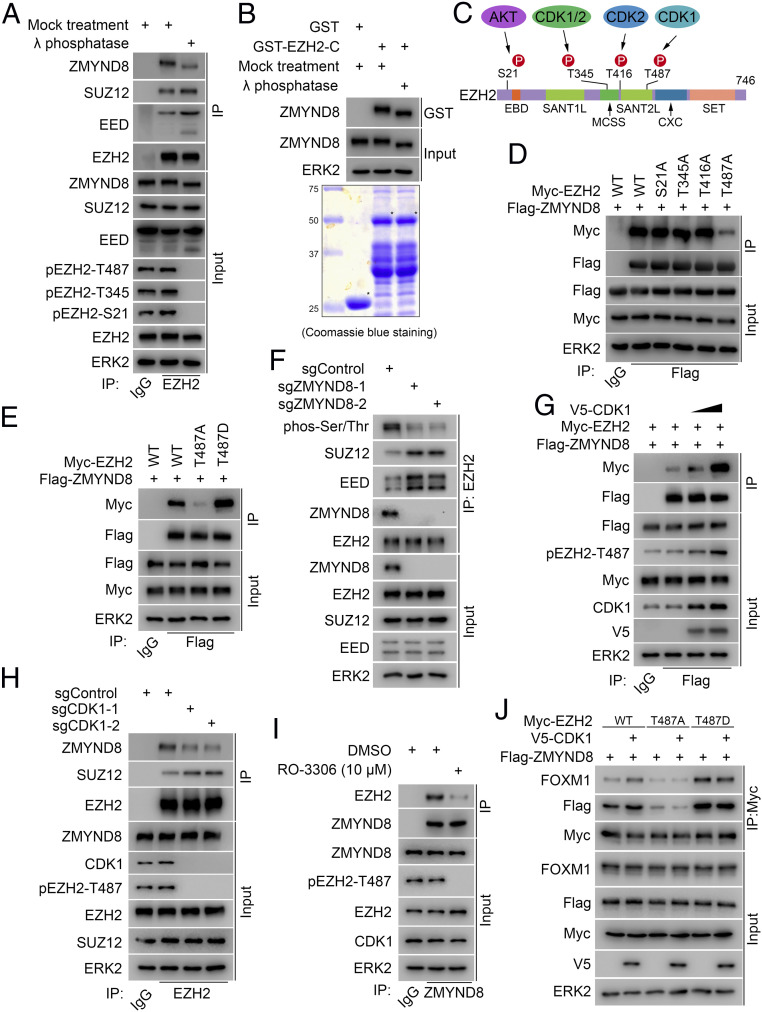
T487 phosphorylation by CDK1 largely enhances EZH2 interaction with ZMYND8. (*A*) WB analysis of co-IP of indicated proteins from 786-O cell lysate treated with or without λ protein phosphatase. (*B*) WB analysis (*Top*) of ZMYND8 from 786-O cell lysate pulled down by GST-EZH2-C recombinant proteins (*Bottom*). (*C*) Schematic diagram showing the EZH2 residues phosphorylated by AKT and CDK1 and CDK2. (*D* and *E*) WB analysis of co-IP of ectopically expressed Flag-ZMYND8 and Myc-EZH2 WT or the indicated mutants in 293T cells. (*F*) WB analysis of EZH2 phosphorylation and co-IP of endogenous EZH2 with SUZ12 and EED in sgControl or sgZMYND8 786-O cells. (*G*) WB analysis of co-IP of ectopically expressed Flag-ZMYND8 and Myc-EZH2 in 293T cells transfected with the indicated plasmids. (*H*) WB analysis of co-IP of endogenous EZH2 with ZMYND8 and SUZ12 in 786-O cells expressing sgControl or sgCDK1. (*I*) WB analysis of co-IP of endogenous ZMYND8 and EZH2 in 786-O cells treated with or without CDK1-selective inhibitor RO-3306 (10 μM; 24 h). (*J*) WB analysis of co-IP of ectopically expressed WT EZH2, T487A or T487D mutant, and/or indicated proteins in 293T cells transfected with indicated plasmids.

Notably, our GST pulldown results (*SI Appendix*, Fig. S3*D*) showed that ZMYND8 binds to EZH2 in the region between amino acids 336 and 554 (*SI Appendix*, Fig. S3*C*), which contains several EZH2 phosphorylation sites (20–22, 33–35). Therefore, we sought to determine whether the ZMYND8–EZH2 interaction is regulated by EZH2 phosphorylation. To this end, we treated 786-O cell lysate with λ protein phosphatase prior to the co-IP assay. As expected, we found that phosphatase treatment blocked EZH2 phosphorylation of several residues, including T487, and increased EZH2 interaction with SUZ12 and EED; however, phosphatase treatment largely decreased the interaction between endogenous ZMYND8 and EZH2 (Fig. 5*A*). We also observed an obvious mobility shift of ZMYND8 protein on Western blot (WB) gels after phosphatase treatment of cell lysate (Fig. 5*A*), suggesting that ZMYND8 is also a phospho-protein. Therefore, we examined whether ZMYND8 phosphorylation plays any role in regulating the binding of these two proteins. We performed GST pulldown assays using GST-EZH2-C recombinant protein, a region interacting with ZMYND8 (*SI Appendix*, Fig. S3*D*). We demonstrated that while phosphatase treatment caused fast mobilization of ZMYND8 protein in both input and GST pulldown samples, it failed to affect ZMYND8 binding with GST-EZH2-C (Fig. 5*B*). These data confirm that phosphorylation of EZH2, but not ZMYND8 protein, is important in regulating ZMYND8–EZH2 interaction.

According to the EZH2 phosphorylation sites reported previously (20–22, 33, 34) (Fig. 5*C*), we mutated those residues individually to alanine (A), including serine 21 (S21A) and threonine residues 345, 416, and 487 (T345A, T416A, and T487A). WT EZH2 and the alanine mutants were transfected into 293T cells for co-IP assays. We demonstrated that different from WT and S21A, T345A and T416A mutants, the binding of T487A mutant to ZMYND8 was largely reduced (Fig. 5*D*). Detection of a residual interaction between ZMYND8 and T487A-mutated EZH2 (Fig. 5*D*) is consistent with the observation that the in vitro translated ZMYND8 is also able to bind to GST-EZH2 recombinant protein purified from bacteria (Fig. 4*E*), suggesting the presence of a certain degree of basal-level constitutive interaction between ZMYND8 and EZH2. We further generated an EZH2 T487 phosphorylation-mimicking mutant (T487D) by converting T487 to aspartic acid (D). We found that the T487D mutation substantially enhanced EZH2 interaction with ZMYND8 (Fig. 5*E*). In addition, ZMYND8 depletion reduced the EZH2 phosphorylation level and increased EZH2 interactions with SUZ12 and EED (Fig. 5*F*), while overexpression of ZMYND8 decreased EZH2 interactions with SUZ12 and EED (*SI Appendix*, Fig. S4*A*), indicating that ZMYND8 interacts with phosphorylated EZH2, which not only protects EZH2 phosphorylation, but also keeps EZH2 away from the PRC2 complex. These results also suggest that EZH2 T487 phosphorylation is important for ZMYND8–EZH2 interaction.

It has been reported that EZH2 T487 is phosphorylated by CDK1 (21). Analysis of the TCGA ccRCC dataset revealed that CDK1 mRNA was significantly elevated in primary human ccRCC tumors compared with adjacent normal kidney tissues (*SI Appendix*, Fig. S4*B*). The CPTAC database (8) also showed that CDK1 expression was much higher in tumors at both mRNA and protein levels compared to their normal counterparts (*SI Appendix*, Fig. S4 *C* and *D*). Overexpression of V5-tagged CDK1 protein substantially increased EZH2 T487 phosphorylation and its interaction with ZMYND8 (Fig. 5*G*). In contrast, knockout of CDK1 by CRISPR/Cas9 in 786-O cells largely decreased EZH2 T487 phosphorylation level and the interaction between endogenous ZMYND8 and EZH2 proteins (Fig. 5*H*) but increased EZH2 interaction with SUZ12, consistent with previously reported results (21). Similar to the results obtained from the genetic manipulation, we found that pharmacologic inhibition by roscovitine, a pan inhibitor of CDK1, CDK2, and CDK5, or RO-3306, a CDK1-selective inhibitor (36, 37), not only abolished EZH2 T487 phosphorylation, but also decreased ZMYND8–EZH2 interaction at the endogenous level in 786-O ccRCC cells (Fig. 5*I* and *SI Appendix*, Fig. S4*E*). Furthermore, we found that while ZMYND8 interaction with WT EZH2 was largely enhanced by CDK1 overexpression, the interaction between ZMYND8 and EZH2 nonphosphorylatable mutant T487A or T487D was unaffected (Fig. 5*J*). These results were confirmed by reciprocal co-IP assays (*SI Appendix*, Fig. S4*F*). Together, these results suggest that CDK1 overexpression enhances EZH2 interaction with ZMYND8, and that this effect is specifically mediated through EZH2 T487 phosphorylation.

### ZMYND8 Is Important for FOXM1 Activation Mediated by EZH2.

**Fig. 6.**
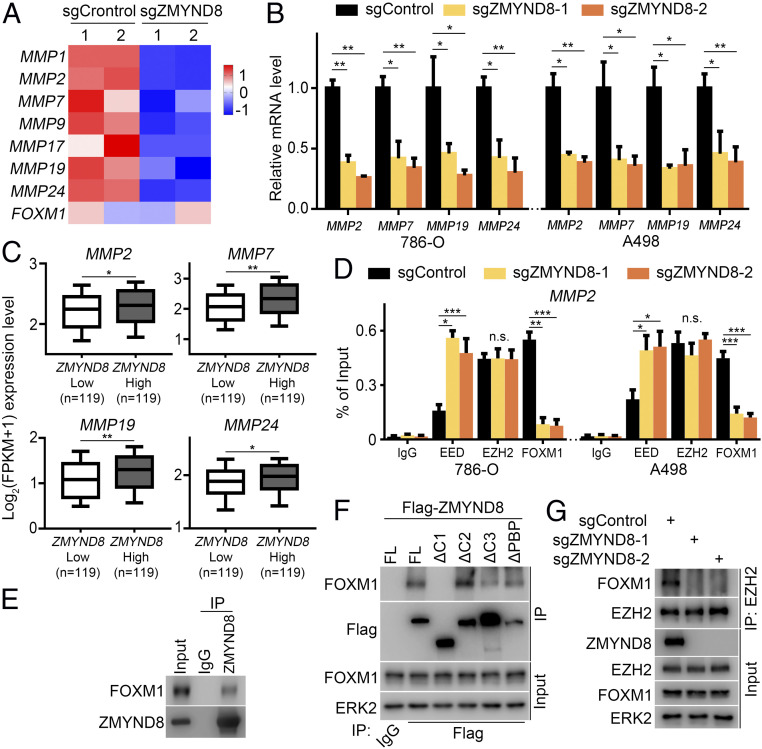
ZMYND8 is important for FOXM1 activation mediated by EZH2. (*A*) Heatmap showing expression of indicated *MMP* genes between sgControl and sgZMYND8 MDA-MB-231 cells under hypoxia. (*B*) sgControl or sgZMYND8 786-O and A498 cells were harvested for RT-qPCR analysis of mRNA expression of the indicated *MMP* genes. *P < 0.05; **P < 0.01. (*C*) Meta-analysis of indicated *MMP* genes at the mRNA level in ZMYND8 high and low TCGA ccRCC patient specimens. *P < 0.05; **P < 0.01. (*D*) sgControl or sgZMYND8 786-O and A498 cells were harvested for ChIP-qPCR analysis of the occupancy of indicated proteins at the *MMP2* gene locus. *P < 0.05; **P < 0.01; ***P < 0.001; n.s., not significant. (*E*) WB analysis of co-IP of endogenous ZMYND8 and FOXM1 in 786-O cells. (*F*) WB analysis of co-IP of FOXM1 with Flag-tagged FL ZMYND8 or truncation mutants in 293T cells. (*G*) WB analysis of co-IP of endogenous EZH2 and FOXM1 in 786-O cells transfected with sgControl or sgZMYND8.

Since our data suggest the presence of a PRC2-independent ZMYND8-EZH2 complex, we further explored the function of this complex. Meta-analysis of RNA-seq data from ZMYND8-proficient and -deficient MDA-MB-231 cells in hypoxic conditions (30) indicated that ZMYND8 depletion inactivates the FOXM1 transcriptional program while activating the Polycomb function of EZH2 (Fig. 1*A*). Notably, it has been reported that HIF activation results in an EZH2 function switch to activating transcription factor FOXM1 (18). Therefore, we sought to explore whether ZMYND8 affects EZH2-FOXM1 function. To this end, we analyzed the RNA-seq data in ZMYND8 WT and KO MDA-MB-231 cancer cells. We found that *MMP* genes, known targets of the EZH2-FOXM1 complex, were down-regulated after ZMYND8 KO in hypoxia-exposed breast cancer cells (Fig. 6*A*). RT-qPCR analysis in 786-O and A498 cells confirmed that expression of several *MMP* genes (*MMP2*, *MMP7*, *MMP19*, and *MMP24*) was indeed down-regulated after ZMYND8 KO (Fig. 6*B*). We further analyzed TCGA RNA-seq data from 238 *VHL*-deleted/truncated ccRCC patients and found that higher ZMYND8 mRNA level positively correlated with higher expression of these *MMP* genes in this cohort of patients (Fig. 6*C*).

It has been reported that PRC2 and the EZH2-FOXM1 complex co-occupy at the *MMP* gene promoters, where they act oppositely in regulating the expression of *MMP* genes (18). Therefore, we performed ChIP-qPCR analysis in 786-O and A498 cells to determine whether ZMYND8 influences PRC2 and EZH2-FOXM1 occupancy on the genomic loci of *MMP* genes. We found that ZMYND8 depletion largely increased PRC2/EED occupancy but decreased FOXM1 binding at the *MMP2* gene promoter, although no EZH2 binding change was detected in the promoter region (Fig. 6*D*). Similar to the findings in breast cancer cells (18), the co-IP assay demonstrated that ZMYND8 and FOXM1 interacted with each other at the endogenous level in ccRCC cells (Fig. 6*E*). Additional co-IP experiments showed that apart from FL ZMYND8 and other deletion mutants (*SI Appendix*, Fig. S3*A*), ZMYND8-ΔC1 failed to bind FOXM1 (Fig. 6*F*), suggesting that a region between PWWP and MYND (amino acids 761 to 1002) in ZMYND8 is required for its interaction with FOXM1.

We further examined whether the ZMYND8–FOXM1 interaction is regulated by phosphorylation. To this end, we treated A498 cell lysate with λ protein phosphatase prior to the co-IP assay. While we observed obvious WB band shifts of both ZMYND8 and FOXM1 proteins after phosphatase treatment, there was no detectable change in the interaction of these two proteins (*SI Appendix*, Fig. S5). Most strikingly, co-IP assay demonstrated that ZMYND8 KO completely abolished the EZH2–FOXM1 interaction, although ZMYND8 depletion had no obvious effect on the expression of either protein (Fig. 6*G*). Furthermore, we found that CDK1 overexpression increased the FOXM1 association with WT EZH2, but not nonphosphorylatable mutants T487A and T487D (Fig. 5*J*). These data suggest that ZMYND8 may function as a scaffold “bridging” the formation of the EZH2-FOXM1 complex.

### ZMYND8 Promotes Tumor Migration and Invasion via Interacting with EZH2 in ccRCC Cells.

**Fig. 7.**
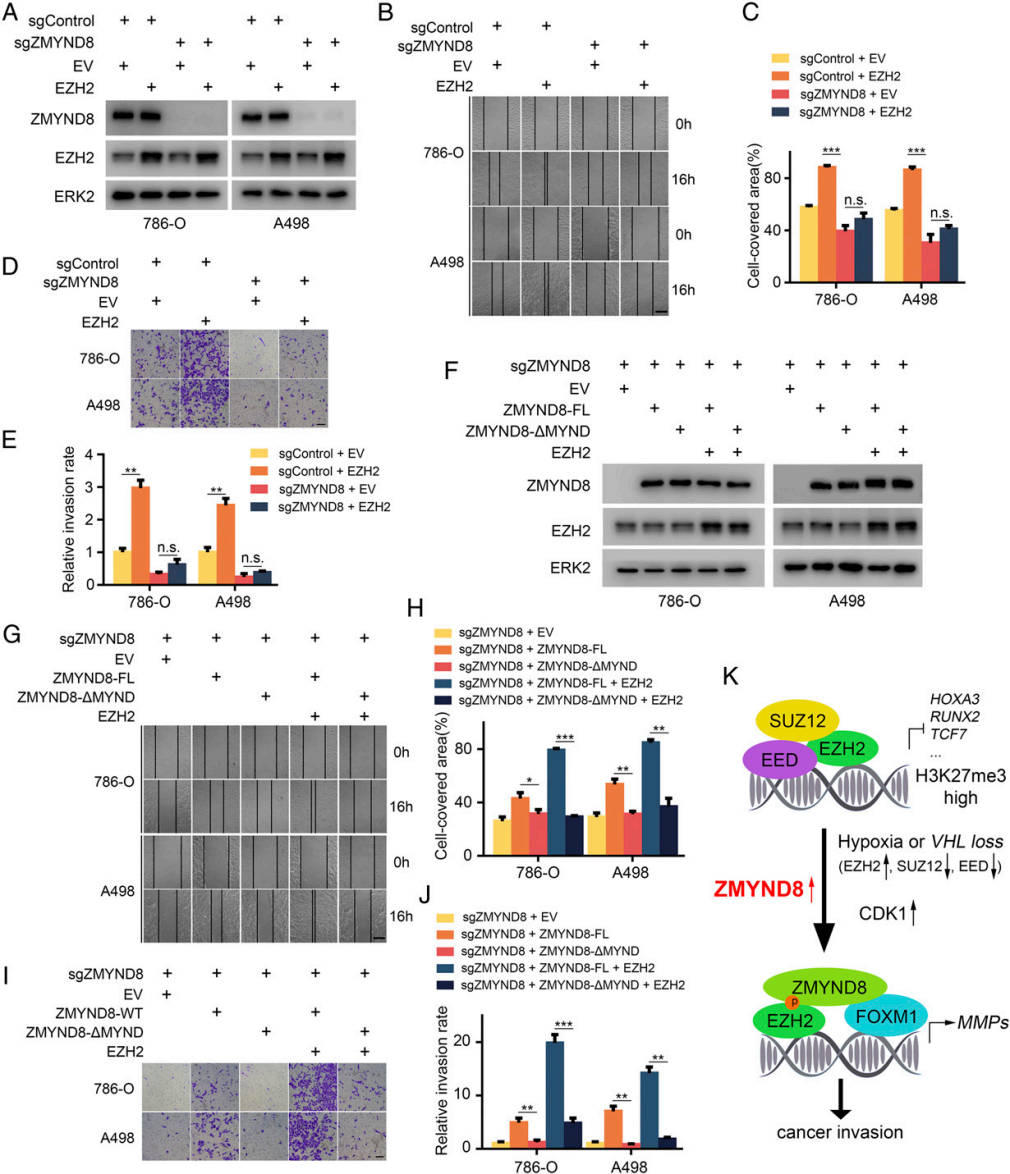
ZMYND8 promotes tumor migration and invasion via interaction with EZH2 in ccRCC cells. (*A*) 786-O and A498 cells stably infected with lentivirus for sgControl or sgZMYND8 were transfected with empty vector (EV) or EZH2. Cells were harvested for WB analysis of indicated proteins. (*B* and *C*) 786-O and A498 stable cell lines were infected with lentivirus as in *A* and cultured to confluence on six-well plates. The cell layer was scratched with a 200-μL pipette tip. For each sample, at least three scratched fields were photographed immediately (0 h) or 16 h after scratching. Photographs of representative images were taken at 100× magnification and results are shown in *B*, and the quantified data are presented in *C*. (Scale bars: 200 μm.) ***P < 0.001; n.s., not significant. (*D* and *E*) 786-O and A498 stable cell lines were infected as in *A* and subjected to Matrigel invasion assays. Representative images of invasion assay are shown in *D*, and the quantified data are presented in *E*. (Scale bars: 200 μm.) **P < 0.01; n.s., not significant. (*F*) sgZMND8 786-O and A498 stable cells were transfected with indicated plasmids, and cells were harvested for WB analysis of indicated proteins. (*G* and *H*) 786-O and A498 stable cell lines were transfected as in *F* and cultured to confluence on six-well plates. The cell layer was scratched with a 200-μL pipette tip. For each sample, at least three scratched fields were photographed immediately (0 h) or 16 h after scratching. Photographs of representative images were taken at 100× magnification and are shown in *G*, and the quantified data are presented in *H*. (Scale bars: 200 μm.) *P < 0.05; **P < 0.01; ***P < 0.001. (*I* and *J*) 786-O and A498 stable cell lines were transfected as in *F* and subjected to Matrigel invasion assays. Representative images of invasion assay are shown in *I*, and the quantified data are presented in *J*. (Scale bars: 200 μm.) **P < 0.01; ***P < 0.001. (*K*) Schematic model depicting the function switch of EZH2 mediated by ZMYND8. In hypoxia-exposed or VHL-deficient cells, EZH2 expression is up-regulated, while other components of PRC2, such as SUZ12 and EED, are down-regulated. Under this condition, overexpressed ZMYND8 preferentially binds to T487-phosphorylated EZH2 mediated by CDK1, which not only is critical for the maintenance of EZH2 phosphorylation and inhibition of assembly of the PRC2 complex, but also mediates EZH2 interaction with FOXM1, thereby promoting FOXM1-dependent expression of *MMP* genes and cancer invasion and progression.

MMPs are major extracellular enzymes involved in cancer invasion and metastasis (38). To elucidate the biological function of the ZMYND8 interaction with EZH2, we performed wound healing and transwell invasion assays to examine their effects on migration and invasion of ccRCC cells. As expected, we found that EZH2 overexpression increased migration and invasion of both 786-O and A498 ccRCC cells (Fig. 7 *A*–*E*). Importantly, these effects were completely abolished by ZMYND8 KO in both cell lines (Fig. 7 *A*–*E*). Moreover, the rescue experiments showed that different from FL ZMYND8, the MYND domain deletion mutant (ZMYND8-ΔMYND) failed to enhance cell migration and invasion (Fig. 7 *F*–*J*), indicating that the MYND domain is important for ZMYND8-mediated cell migration and invasion. Similarly, this domain is also critical for the cooperative effects of EZH2 and ZMYND8 on migration and invasion in these two cell lines (Fig. 7 *F*–*J*). These results indicate that interaction with ZMYND8 plays a pivotal role in EZH2-mediated migration and invasion in ccRCC cells.

## Discussion

ZMYND8 was originally identified in a protein complex possessing histone deacetylase and nucleosome remodeling activities (39, 40). ZMYND8 canonically functions as a chromatin reader that recognizes histone codes, such as methylation (H3K4me1, H3K4me3, and H3K36me2) (25, 26, 28) and/or acetylation (H4K14ac and H4K16ac) (25, 28). In the present study, we demonstrate that ZMYND8 preferentially binds phosphorylated nonhistone protein EZH2. Moreover, the histone reader functions of ZMYND8 are shown to be mediated by the BROMO and PWWP domains (27, 28). In contrast, the distinct EZH2 phosphorylation recognition activity of ZMYND8 is dependent on the MYND domain in a manner independent of the BROMO and PWWP domains. Thus, in addition to recognizing histone codes such as methylation and acetylation, we reveal a reader function of ZMYND8 in recognizing a phosphorylation “code” on a nonhistone protein. Moreover, along with binding of histone deacetylases, ZMYND8 is also associated with other histone “erasers,” such as demethylases, including KDM5A, KDM5C, and LSD1 (26, 27, 41, 42). Our results extend previously reported findings by identifying the epigenetic “writer” EZH2 methyltransferase as a binding partner of ZMYND8.

EZH2 canonically acts as a gene repressor in a PRC2-dependent manner by catalyzing H3K27me3 to mediate gene silencing (9–12). Notably, the PRC2 complex is disassembled due to EZH2 phosphorylation at different residues, such as S21 and T487, mediated by AKT and CDK1 kinases, respectively (20, 21). Further studies have shown that EZH2 phosphorylation at these sites not only abolishes its Polycomb-dependent repressor function, but also allows EZH2 to gain a Polycomb-independent gene activator function (19). Our present findings show that ZMYND8 can bind to EZH2 in a manner largely dependent on T487 phosphorylation mediated by CDK1. We further show that ZMYND8 functions as a scaffold protein that is critical for mediating the EZH2 recruitment of FOXM1 and the expression of cancer migration and invasion genes transactivated by the EZH2-FOXM1 complex. Additionally, it seems that ZMYND8 binding also protects and maintains EZH2 phosphorylation at T487, thereby tipping EZH2 function away from PRC2 and toward Polycomb-independent functions. Thus, our current findings advance the field by identifying an additional layer of regulation of EZH2 phosphorylation and associated tumor biology.

Notably, we observed that ZMYND8 antagonizes Polycomb-dependent function to inhibit H3K27me3 level only in hypoxia- or VHL-deficient cancer cells, conditions of which are commonly associated with the up-regulation of HIF transcription factors. In agreement with a previous report showing that HIF1α inhibits PRC2 activities (18), we confirm that EZH2 expression is substantially up-regulated while SUZ12 and EED, two key components of PRC2 complex, are largely down-regulated in hypoxia- or VHL-deficient cells. Based on our findings, we envision a model wherein under hypoxic or VHL-deficient (but normoxic) conditions, overexpression of ZMYND8 cooperates with CDK1 to promote EZH2 T487 phosphorylation-dependent formation of an EZH2-FOXM1 complex by leveraging the extra “free” EZH2 proteins that are unassociated with the PRC2 complex. As a result, ZMYND8 overexpression impairs EZH2 Polycomb-dependent gene repressor function but activates EZH2 Polycomb-independent gene activator function, thereby enhancing cancer migration and invasion (Fig. 7*K*).

In summary, our findings uncover a previously unidentified role of ZMYND8 in regulating PRC2 activities and promoting a Polycomb-dependent to -independent switch of EZH2 functions in hypoxia-exposed or VHL-deficient cancer cells. Our data suggest that EZH2 T487 phosphorylation is important for ZMYND8–EZH2 interaction, and that this interaction can be largely enhanced by CDK1 phosphorylation of EZH2. We also demonstrate the ZMYND8 is essential in mediating the EZH2–FOXM1 interaction and *MMP* gene induction. Thus, this essential role of ZMYND8 in regulating the gene activator function of EZH2 might represent a viable target for the effective treatment of cancers under hypoxia or with VHL deficiency, such as ccRCC.

## Materials and Methods

The materials used in this study, including cell lines, chemicals, antibodies, and sequences for sgRNAs, siRNAs, RT-qPCR and ChIP primers, are described in *SI Appendix*. TCGA ccRCC patient data were generated using the web-based tools from UCSC Xena (https://xena.ucsc.edu/) and cBioPortal (https://www.cbioportal.org/). CPTAC ccRCC patient data were derived through a meta-analysis from the CPTAC Data Portal (https://cptac-data-portal.georgetown.edu/). Detailed descriptions of the study methods, including cell culture and transfection, stable cell line generation, TMA, IHC, co-IP, WB, GST pulldown assays, RT-qPCR and ChIP-qPCR, and in vitro migration and invasion assays, are also provided in *SI Appendix*.

## Supplementary Material

Supplementary File

## Data Availability

All study data are included in the main text and *SI Appendix*.

## References

[1] G. L. Semenza, Oxygen sensing, hypoxia-inducible factors, and disease pathophysiology. Annu. Rev. Pathol. 9, 47–71 (2014). 23937437 10.1146/annurev-pathol-012513-104720

[2] W. G. Kaelin Jr, The von Hippel-Lindau tumour suppressor protein: O_2_ sensing and cancer. Nat. Rev. Cancer 8, 865–873 (2008). 18923434 10.1038/nrc2502

[3] L. Schito, G. L. Semenza, Hypoxia-inducible factors: Master regulators of cancer progression. Trends Cancer 2, 758–770 (2016). 28741521 10.1016/j.trecan.2016.10.016

[4] Y. Z. Gu, S. M. Moran, J. B. Hogenesch, L. Wartman, C. A. Bradfield, Molecular characterization and chromosomal localization of a third alpha-class hypoxia inducible factor subunit, HIF3alpha. Gene Expr. 7, 205–213 (1998). 9840812 PMC6151950

[5] H. Tian, S. L. McKnight, D. W. Russell, Endothelial PAS domain protein 1 (EPAS1), a transcription factor selectively expressed in endothelial cells. Genes Dev. 11, 72–82 (1997). 9000051 10.1101/gad.11.1.72

[6] G. L. Wang, B. H. Jiang, E. A. Rue, G. L. Semenza, Hypoxia-inducible factor 1 is a basic-helix-loop-helix-PAS heterodimer regulated by cellular O_2_ tension. Proc. Natl. Acad. Sci. U.S.A. 92, 5510–5514 (1995). 7539918 10.1073/pnas.92.12.5510 PMC41725

[7] A. C. Epstein., C. elegans EGL-9 and mammalian homologs define a family of dioxygenases that regulate HIF by prolyl hydroxylation. Cell 107, 43–54 (2001). 11595184 10.1016/s0092-8674(01)00507-4

[8] D. J. Clark.; Clinical Proteomic Tumor Analysis Consortium, Integrated proteogenomic characterization of clear cell renal cell carcinoma. Cell 180, 207 (2020). 31923397 10.1016/j.cell.2019.12.026

[9] R. Cao., Role of histone H3 lysine 27 methylation in Polycomb group silencing. Science 298, 1039–1043 (2002). 12351676 10.1126/science.1076997

[10] B. Czermin., Drosophila enhancer of Zeste/ESC complexes have a histone H3 methyltransferase activity that marks chromosomal Polycomb sites. Cell 111, 185–196 (2002). 12408863 10.1016/s0092-8674(02)00975-3

[11] A. Kuzmichev, K. Nishioka, H. Erdjument-Bromage, P. Tempst, D. Reinberg, Histone methyltransferase activity associated with a human multiprotein complex containing the Enhancer of Zeste protein. Genes Dev. 16, 2893–2905 (2002). 12435631 10.1101/gad.1035902 PMC187479

[12] J. Müller., Histone methyltransferase activity of a Drosophila polycomb group repressor complex. Cell 111, 197–208 (2002). 12408864 10.1016/s0092-8674(02)00976-5

[13] T. H. Ho., Multicenter validation of enhancer of zeste homolog 2 expression as an independent prognostic marker in localized clear cell renal cell carcinoma. J. Clin. Oncol. 35, 3706–3713 (2017). 28976794 10.1200/JCO.2017.73.3238 PMC5678341

[14] C. G. Kleer., EZH2 is a marker of aggressive breast cancer and promotes neoplastic transformation of breast epithelial cells. Proc. Natl. Acad. Sci. U.S.A. 100, 11606–11611 (2003). 14500907 10.1073/pnas.1933744100 PMC208805

[15] S. Varambally., The polycomb group protein EZH2 is involved in progression of prostate cancer. Nature 419, 624–629 (2002). 12374981 10.1038/nature01075

[16] E. Kim., Phosphorylation of EZH2 activates STAT3 signaling via STAT3 methylation and promotes tumorigenicity of glioblastoma stem-like cells. Cancer Cell 23, 839–852 (2013). 23684459 10.1016/j.ccr.2013.04.008 PMC4109796

[17] S. T. Lee., Context-specific regulation of NF-κB target gene expression by EZH2 in breast cancers. Mol. Cell 43, 798–810 (2011). 21884980 10.1016/j.molcel.2011.08.011

[18] S. Mahara., HIFI-α activation underlies a functional switch in the paradoxical role of Ezh2/PRC2 in breast cancer. Proc. Natl. Acad. Sci. U.S.A. 113, E3735–E3744 (2016). 27303043 10.1073/pnas.1602079113 PMC4932959

[19] K. Xu., EZH2 oncogenic activity in castration-resistant prostate cancer cells is Polycomb-independent. Science 338, 1465–1469 (2012). 23239736 10.1126/science.1227604 PMC3625962

[20] T. L. Cha., Akt-mediated phosphorylation of EZH2 suppresses methylation of lysine 27 in histone H3. Science 310, 306–310 (2005). 16224021 10.1126/science.1118947

[21] Y. Wei., CDK1-dependent phosphorylation of EZH2 suppresses methylation of H3K27 and promotes osteogenic differentiation of human mesenchymal stem cells. Nat. Cell Biol. 13, 87–94 (2011). 21131960 10.1038/ncb2139 PMC3076036

[22] L. Wan., Phosphorylation of EZH2 by AMPK suppresses PRC2 methyltransferase activity and oncogenic function. Mol. Cell 69, 279–291.e5 (2018). 29351847 10.1016/j.molcel.2017.12.024 PMC5777296

[23] W. K. Bae, L. Hennighausen, Canonical and non-canonical roles of the histone methyltransferase EZH2 in mammary development and cancer. Mol. Cell. Endocrinol. 382, 593–597 (2014). 23684884 10.1016/j.mce.2013.05.002 PMC3843995

[24] L. Dong., 35H, a sequence isolated as a protein kinase C binding protein, is a novel member of the adducin family. J. Biol. Chem. 270, 25534–25540 (1995). 7592723 10.1074/jbc.270.43.25534

[25] N. Li., ZMYND8 reads the dual histone mark H3K4me1-H3K14ac to antagonize the expression of metastasis-linked genes. Mol. Cell 63, 470–484 (2016). 27477906 10.1016/j.molcel.2016.06.035 PMC4975651

[26] H. Shen., Suppression of enhancer overactivation by a RACK7-histone demethylase complex. Cell 165, 331–342 (2016). 27058665 10.1016/j.cell.2016.02.064 PMC4826479

[27] F. Gong., Screen identifies bromodomain protein ZMYND8 in chromatin recognition of transcription-associated DNA damage that promotes homologous recombination. Genes Dev. 29, 197–211 (2015). 25593309 10.1101/gad.252189.114 PMC4298138

[28] S. Adhikary., Selective recognition of H3.1K36 dimethylation/H4K16 acetylation facilitates the regulation of all-trans-retinoic acid (ATRA)-responsive genes by putative chromatin reader ZMYND8. J. Biol. Chem. 291, 2664–2681 (2016). 26655721 10.1074/jbc.M115.679985 PMC4742736

[29] F. Jiao., RACK7 recognizes H3.3G34R mutation to suppress expression of MHC class II complex components and their delivery pathway in pediatric glioblastoma. Sci. Adv. 6, eaba2113 (2020). 32832624 10.1126/sciadv.aba2113 PMC7439511

[30] Y. Chen., ZMYND8 acetylation mediates HIF-dependent breast cancer progression and metastasis. J. Clin. Invest. 128, 1937–1955 (2018). 29629903 10.1172/JCI95089 PMC5919820

[31] J. A. Simon, C. A. Lange, Roles of the EZH2 histone methyltransferase in cancer epigenetics. Mutat. Res. 647, 21–29 (2008). 18723033 10.1016/j.mrfmmm.2008.07.010

[32] P. Savitsky., Multivalent histone and DNA engagement by a PHD/BRD/PWWP triple reader cassette recruits ZMYND8 to K14ac-rich chromatin. Cell Rep. 17, 2724–2737 (2016). 27926874 10.1016/j.celrep.2016.11.014 PMC5177622

[33] C. C. Yang., Phosphorylation of EZH2 at T416 by CDK2 contributes to the malignancy of triple-negative breast cancers. Am. J. Transl. Res. 7, 1009–1020 (2015). 26279746 PMC4532735

[34] S. Chen., Cyclin-dependent kinases regulate epigenetic gene silencing through phosphorylation of EZH2. Nat. Cell Biol. 12, 1108–1114 (2010). 20935635 10.1038/ncb2116 PMC3292434

[35] X. Zeng, S. Chen, H. Huang, Phosphorylation of EZH2 by CDK1 and CDK2: A possible regulatory mechanism of transmission of the H3K27me3 epigenetic mark through cell divisions. Cell Cycle 10, 579–583 (2011). 21278485 10.4161/cc.10.4.14722 PMC3174000

[36] S. Hellmuth, L. Gómez-H, A. M. Pendás, O. Stemmann, Securin-independent regulation of separase by checkpoint-induced shugoshin-MAD2. Nature 580, 536–541 (2020). 32322060 10.1038/s41586-020-2182-3

[37] L. T. Vassilev., Selective small-molecule inhibitor reveals critical mitotic functions of human CDK1. Proc. Natl. Acad. Sci. U.S.A. 103, 10660–10665 (2006). 16818887 10.1073/pnas.0600447103 PMC1502288

[38] Q. Yao, L. Kou, Y. Tu, L. Zhu, MMP-responsive “smart” drug delivery and tumor targeting. Trends Pharmacol. Sci. 39, 766–781 (2018). 30032745 10.1016/j.tips.2018.06.003

[39] J. K. Tong, C. A. Hassig, G. R. Schnitzler, R. E. Kingston, S. L. Schreiber, Chromatin deacetylation by an ATP-dependent nucleosome remodelling complex. Nature 395, 917–921 (1998). 9804427 10.1038/27699

[40] Y. Zhang, G. LeRoy, H. P. Seelig, W. S. Lane, D. Reinberg, The dermatomyositis-specific autoantigen Mi2 is a component of a complex containing histone deacetylase and nucleosome remodeling activities. Cell 95, 279–289 (1998). 9790534 10.1016/s0092-8674(00)81758-4

[41] A. Malovannaya., Analysis of the human endogenous coregulator complexome. Cell 145, 787–799 (2011). 21620140 10.1016/j.cell.2011.05.006 PMC3131083

[42] H. C. Eberl, C. G. Spruijt, C. D. Kelstrup, M. Vermeulen, M. Mann, A map of general and specialized chromatin readers in mouse tissues generated by label-free interaction proteomics. Mol. Cell 49, 368–378 (2013). 23201125 10.1016/j.molcel.2012.10.026

