# Supplementary material for: ZMYND8 preferentially binds phosphorylated EZH2 to promote a PRC2-dependent to -independent function switch in hypoxia-inducible factor–activated cancer
Source: Proc Natl Acad Sci U S A. 2021 Feb 15;118(8):e2019052118. doi: 10.1073/pnas.2019052118 (PMC7923384; doi:10.1073/pnas.2019052118)
Supplement: Supplementary File [file pnas.2019052118.sapp.pdf]

PNAS
www.pnas.org

Supplementary Information for

ZMYND8 preferentially binds phosphorylated EZH2 to promote PRC2-dependent-to-independent function switch in HIF-activated cancer

Bo Tang[1,2], Rui Sun[2], Dejie Wang[2], Haoyue Sheng[2,3,4], Ting Wei[5], Liguo Wang[5], Jun Zhang[6], Thai H. Ho[7], Lu Yang[1], Qiang Wei[1]* and Haojie Huang[2,8,9]*

[1]Department of Urology, Institute of Urology, West China Hospital, Sichuan University, Chengdu 610041, China

[2]Department of Biochemistry and Molecular Biology, Mayo Clinic College of Medicine and Science, Rochester, MN 55905, USA

[3]Department of Urology, Fudan University Shanghai Cancer Center, Shanghai 200032, China

[4]Department of Oncology, Shanghai Medical College, Fudan University, Shanghai 200032, China

[5]Division of Biomedical Statistics and Informatics, Mayo Clinic College of Medicine and Science, Rochester, MN 55905, USA

[6]Department of Laboratory Medicine and Pathology, Mayo Clinic College of Medicine and Science, Scottsdale, AZ 85259, USA

[7]Division of Hematology and Oncology, Department of Internal Medicine, Mayo Clinic College of Medicine and Science, Phoenix, AZ 85054, USA

[8]Department of Urology, Mayo Clinic College of Medicine and Science, Rochester, MN 55905, USA

[9]Mayo Clinic Cancer Center, Mayo Clinic College of Medicine and Science, Rochester, MN 55905, USA

***Corresponding authors**: Haojie Huang, Ph.D. (huang.haojie@mayo.edu); Qiang Wei, M.D. (weiqiang163163@163.com)

This PDF file includes:

Supplementary text

Figure S1 to S5

Table S1 to S4

Supplementary text

Supplementary Methods (SI Methods)

Cell culture and transfection

786-O, A498, 293T and MDA-MB-231 cell lines were purchased from American Type Culture Collection (ATCC). RCC10 cell line was kindly provided by Dr. Jun Liu at Mayo Clinic. A498, RCC10, 293T and MDA-MB-231 cells were maintained in DMEM supplemented with 10% FBS. 786-O cells were cultured in RPMI 1640 supplemented with 10% FBS. Cells were cultured at 37℃ supplied with 5% CO2. Mycoplasma contamination was regularly examined using Lookout Mycoplasma PCR Detection Kit (Sigma-Aldrich). Plasmocin (InvivoGen) was routinely added to the cell culture medium to prevent or eliminate mycoplasma contamination. Transfections were performed by using Lipofectamine 2000 (Thermo Fisher Scientific). Approximately 75% - 95% transfection efficiencies were routinely achieved.

Stable cell line generation

Lentivirus transduction system was utilized to generate stable cell lines with specific gene knockdown or overexpression. PEI was used to transfect sgRNA plasmids together with lentivirus package plasmids (PSPAX2 and PMD2.G) into 293T cells. 48 h after transfection, supernatant containing viruses was collected, filtered and utilized to infect indicated cells. Polybrene (8 mg/ml) was added to the viral supernatant to increase the infection efficiency. 48 h after infection, culture medium was replaced with fresh medium, and puromycin (1.5 mg/ml) was administrated for cell selection. sgRNA sequence information is provided in *SI Appendix* Table S1.

Tissue microarray (TMA) and immunohistochemistry (IHC)

The TMA slides were purchased from US Biolab (Catalogue no. KID501). TMA were deparaffinized, rehydrated and subjected to heat-mediated antigen retrieval. The UltraSensitive S-P (Rabbit) IHC Kit (KIT-9706, Fuzhou Maixin Biotech) was used following the manufacturer's instructions with minor modifications. Briefly, sections were incubated with 3% H2O2 for 15 min at room temperature to quench endogenous peroxidase activity. After antigen retrieval using unmasking solution (Vector Labs), slides were blocked with normal goat serum for 1 h and then incubated with primary antibody at 4 °C overnight. IHC analysis of tumor samples was performed using primary anti-ZMYND8 and anti-H3K27me3 rabbit antibodies. The sections were then washed three times in 1× PBS and treated for 30 min with biotinylated goat anti-rabbit IgG secondary antibodies (Fuzhou Maixin Biotech). After washing three times in 1× PBS, sections were incubated with streptavidin-conjugated HRP (Fuzhou Maixin Biotech). After washing three times in 1× PBS for 5 min each, specific detection was developed with 3,3′-diaminobenzidine (DAB-2031, Fuzhou Maixin Biotech). Images were acquired using Leica DM2000 microscope and matched software. The IHC staining was evaluated by two independent investigators including one genitourinary cancer pathologist who were blinded to the clinical details. Based on the percentage of positive cells and the staining intensity, the final immunoreactivity score (IS) for each case was calculated as follows: staining percentage × intensity.

Co-immunoprecipitation (co-IP) and Western blot (WB)

For co-IP assay, cells were lysed with IP buffer (150 mM NaCl, 50 mM Tris-HCl pH = 7.5, 1%

Nonidet P-40, 0.5% sodium deoxycholate and 1% protease inhibitor cocktails) for 30 min, and cell lysate was harvested by centrifuging followed by incubation with indicated antibodies and Protein G Plus/Protein A agarose beads (Sigma-Aldrich, USA) at 4°C overnight. Next day, the beads bound by target proteins were washed 6 times with IP buffer. Proteins were denatured for WB analysis. For WB, target proteins were denatured using sample buffer suppled with 10% DTT (Thermo Fisher Scientific, USA) and denatured at 95°C for 10 min. Samples were subjected to SDS-polyacrylamide gel (Bio-Rad, USA) separation, and the gels were further transferred to nitrocellulose (NC) membranes (Thermo Fisher Scientific, USA). After transferring, the NC membranes were blocked in 5% non-fat milk (Bio-Rad, USA) for 1 h at room temperature and incubated with the indicated primary antibodies at 4°C overnight. Next day, the NC membranes were washed with TBST for 10 min each for three times and incubated with matched secondary antibody for 1 h at room temperature. The membranes were washed with TBST for 10 min each for three more times. The protein was visualized by SuperSignal West Pico Stable Peroxide Solution (Thermo Fisher Scientific). Antibody information can be found in *SI Appendix* Table S2.

Glutathione S-transferase (GST) pulldown assay

Cells were lysed with IP buffer (50 mM Tris-HCl, pH 7.4, 150 mM NaCl, 1% Triton X-100, 1% sodium deoxycholate and 1% protease inhibitor cocktails) on ice for more than 30 min. GST fusion proteins were immobilized on glutathione-Sepharose beads (GE Healthcare Lifesciences). After washing with lysis buffer, the beads were incubated with cell lysates or proteins generated using the TNT quick coupled transcript/translation system (Progema) at 4°C overnight. The beads were then washed six times with binding buffer and re-suspended in sample buffer. The

bound proteins were subjected to WB analysis.

RT-qPCR and ChIP-qPCR

Total RNA was extracted with TRIzol (Invitrogen) and reverse transcribed into cDNA with SuperScript III First-Strand Synthesis System (Promega). Quantitative PCR was performed in the iQ thermal cycler (Bio-Rad) using the iQ SYBR Green Supermix (Bio-Rad) and in triplicate. The ΔCT was calculated by normalizing the threshold difference of certain gene with glyceraldehyde-3-phosphate dehydrogenase (*GAPDH*). Sequence information for primers used for RT-qPCR is provided in *SI Appendix* Table S3.

ChIP-qPCR was performed as previously described(1). Briefly, formaldehyde (11%) solution was utilized to crosslink chromatin in cells for 10 min at room temperature. Crosslinked chromatin was sonicated, and immunoprecipitated with Protein G Plus/Protein A agarose beads (Sigma-Aldrich, USA) together with indicated antibody at 4°C overnight. The Protein-DNA complexes were precipitated and eluted and cross-linking was reversed at 65°C for 16 h. DNA fragments were purified and analyzed by real-time PCR. Sequence information for primers used for ChIP-qPCR is provided in *SI Appendix* Table S4.

In vitro migration assay

Cells were transfected with the indicated plasmids and cultured to confluence on 6-well plates. The cell layer was scratched with a 200-μl pipette tip, and detached cells were removed. For each sample, at least three scratched fields were photographed immediately. Cell migration was evaluated by measuring the cell-covered area.

In vitro invasion assay

The in vitro cell invasion assay was performed using a Bio-Coat Matrigel invasion chamber (BD Biosciences) according to the protocol of the manufacturer. 786-O and A498 cells were cultured in the insert for 24 h. Cells were fixed in methanol for 15 min and then stained with 1 mg/ml crystal violet for 20 min. At least five fields for each group were photographed after staining, and invaded cells were counted.

Statistics analysis

All data are expressed as means±S.D. For experiments with only two groups, Student's t test was used for statistical comparisons. For analysis of correlation between ZMYND8 and H3K27me3 protein expression in human ccRCC specimens, Pearson's product-moment correlation was used. $P < 0.05$ was considered statistically significant.

References for SI Methods

1. Yang Y, *et al.* (2017) Loss of FOXO1 Cooperates with TMPRSS2-ERG Overexpression to Promote Prostate Tumorigenesis and Cell Invasion. *Cancer Res* 77(23):6524-6537.

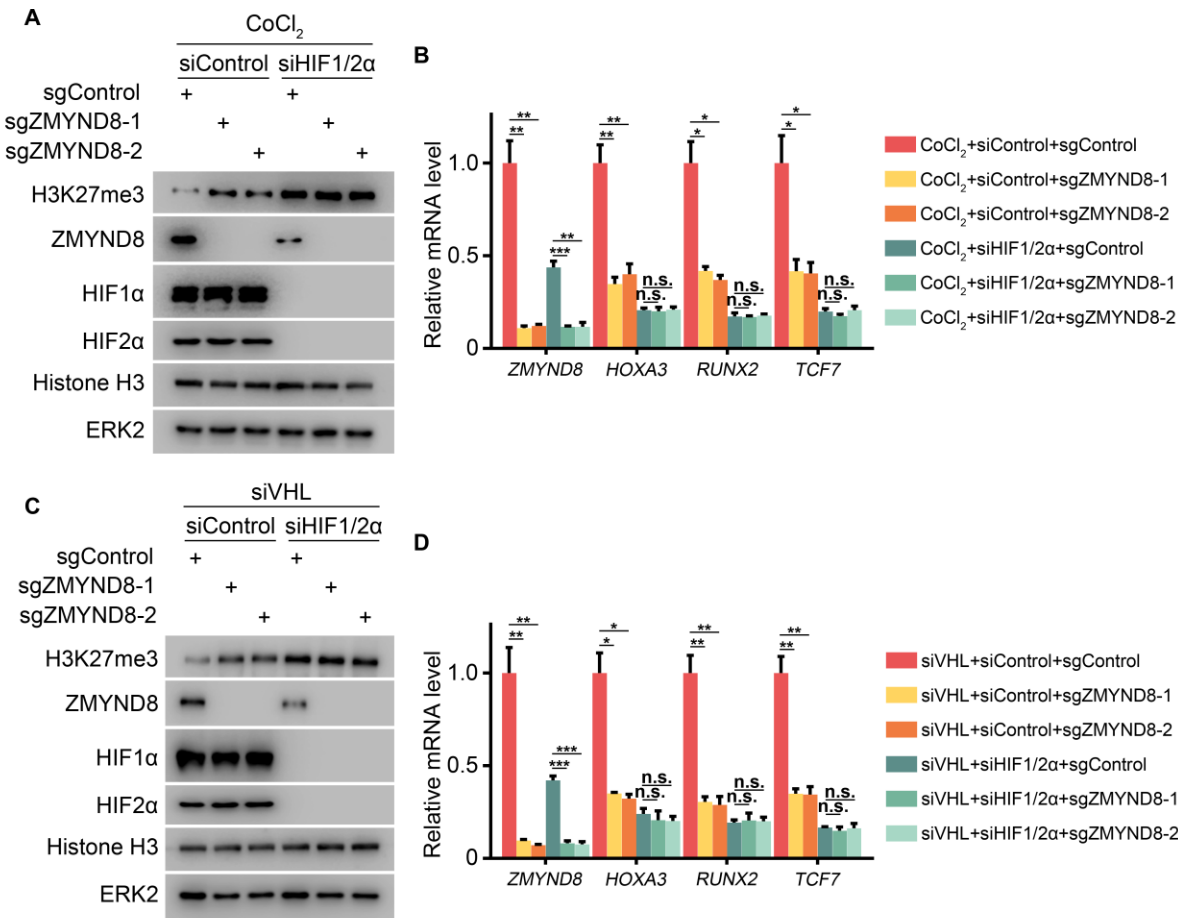

Fig S1. ZMYND8 negatively regulates PRC2 target gene expression in breast cancer cells under hypoxia (related to Fig. 1).

(*A* and *B*) MDA-MB-231 cells stably expressing control (sgControl) or ZMYND8-specific sgRNAs (sgZMYND8-1 or sgZMYND8-2) were transfected with indicated siRNAs (siControl or siHIF1/2α) for 48 h and treated with hypoxia mimetic reagent $CoCl_2$ (200 μM) for 24 h. Cells were harvested for WB for indicated proteins (*A*) and RT-qPCR analysis of mRNA expression of the indicated genes (*B*). $^*P < 0.05$, $^{**}P < 0.01$, $^{***}P < 0.001$, n.s., not significant.

(*C* and *D*) MDA-MB-231 cells stably expressing control (sgControl) or ZMYND8-specific sgRNAs (sgZMYND8-1 or sgZMYND8-2) were transfected with indicated siRNAs (siControl,

siHIF1/2α and/or siVHL) for 48 h and harvested for WB of indicated proteins (*C*) and RT-qPCR analysis of mRNA expression of the indicated genes (*D*). $^{*}P < 0.05$, $^{**}P < 0.01$, $^{***}P < 0.001$, n.s., not significant.

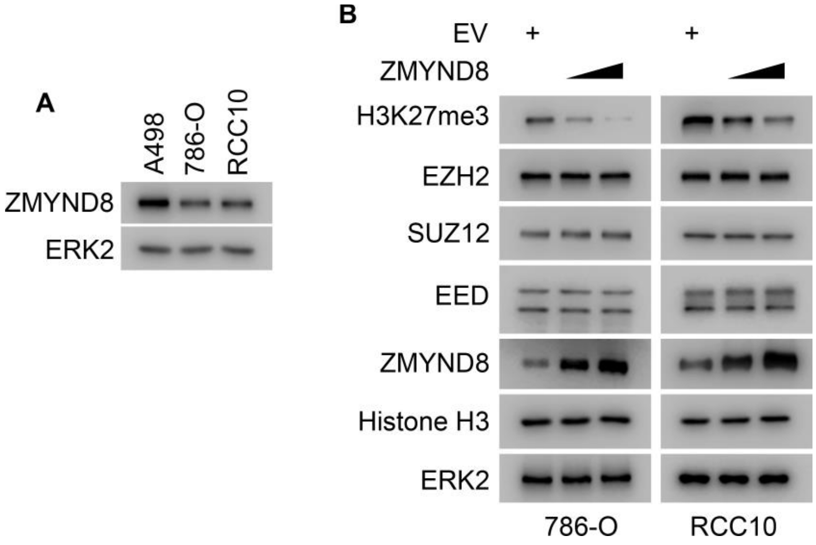

Fig S2. ZMYND8 negatively regulates H3K27me3 target gene expression in ccRCC cells (related to Fig. 3).

(*A*) WB analysis of ZMYND8 protein level in different ccRCC cell lines. ERK2 was used as a loading control.

(*B*) WB analysis of the indicated proteins in lysate of 786-O and A498 cells transfected with empty vector (EV) or ZMYND8.

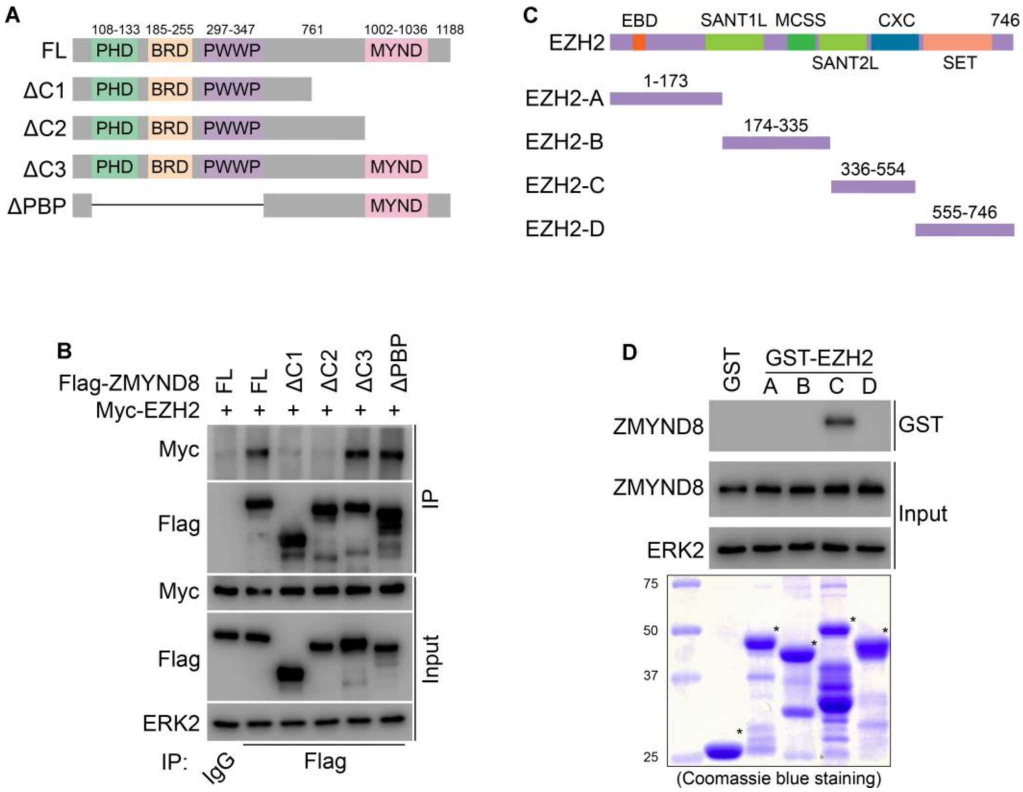

Fig S3. ZMYND8 interacts with EZH2 in ccRCC cells (related to Fig. 4).

(*A*) Schematic diagram showing Flag-tagged full-length (FL) and domain deletion constructs of ZMYND8.

(*B*) WB analysis of co-IP of Myc-EZH2 with Flag-tagged FL ZMYND8 and truncation mutants in 293T cells.

(*C*) Schematic diagram depicting a set of GST-EZH2 recombinant protein constructs.

(*D*) WB analysis of ZMYND8 proteins from 786-O whole cell lysate pulled down by GST or GST-EZH2 recombinant proteins.

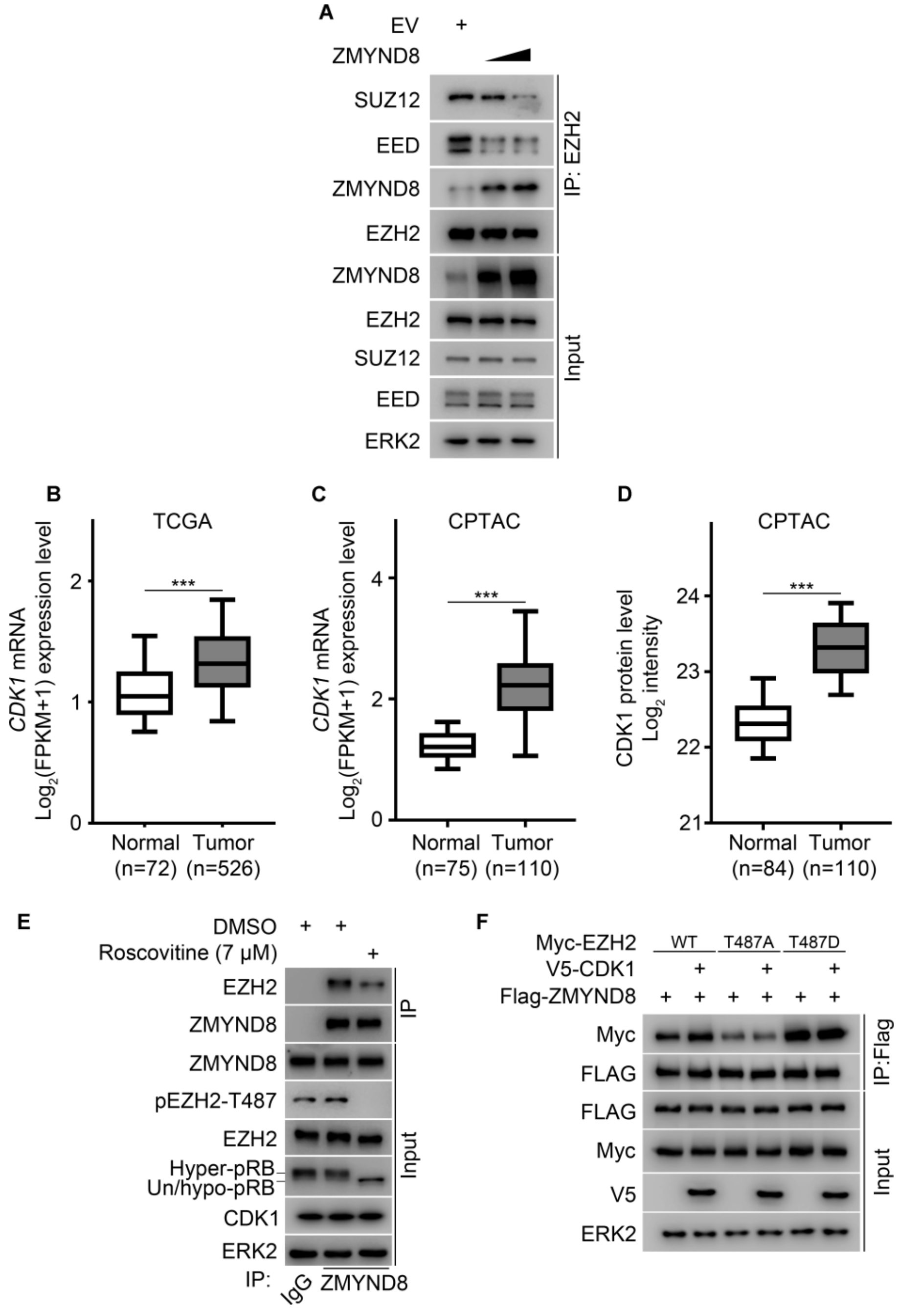

Fig S4. T487 phosphorylation by CDK1 largely enhances EZH2 interaction with ZMYND8 (related to Fig. 5).

(*A*) WB analysis of co-IP of endogenous EZH2 with SUZ12 and EED in RCC10 cells transfected

with empty vector (EV) or increased doses of ZMYND8.

(*B*) Meta-analysis of *CDK1* mRNA level in the TCGA cohort of normal and ccRCC patient specimens. ***$P < 0.001$.

(*C*) Meta-analysis of *CDK1* mRNA level in the CPTAC cohort of normal and ccRCC patient specimens. ***$P < 0.001$.

(*D*) Meta-analysis for CDK1 protein level in the CPTAC cohort of normal and ccRCC patient specimens. ***$P < 0.001$.

(*E*) WB analysis of co-IP of endogenous ZMYND8 and EZH2 in 786-O cells treated with or without Roscovitine. RB protein phosphorylation as a positive control.

(*F*) WB analysis of co-IP of ectopically expressed Flag-ZMYND8 and will-type (WT) EZH2 and the indicated mutants in 293T cells transfected with indicated plasmids.

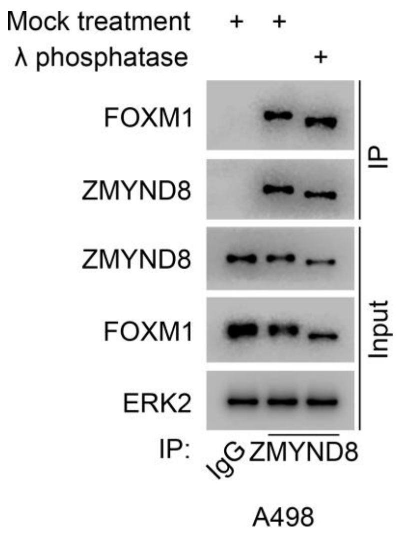

Fig S5. Impact of protein phosphorylation on ZMYND8 and FOXM1 interaction (related to Fig. 6).

WB analysis of co-IP of indicated proteins from A498 cell lysate treated with or without λ protein phosphatase.

Table S1. Oligonucleotide sequences of sgRNAs and siRNAs

sgZMYND8-1	GACTTAGCGTGATAAACCCG
sgZMYND8-2	GGAGCGCGGCATATCCGACA
sgCDK1-1	ACACAATCCCCTGTAGGATT
sgCDK1-2	GAATCCATGTACTGACCAGG
siVHL-1	ACACAGGAGCGCATTGCACAT
siVHL-2	AAGAGTACGGCCCTGAAGAAG
siHIF1A	AACTGATGACCAGCAACTTGA
siEPAS1/siHIF2α	AACAGCATCTTTGATAGCAGT

Table S2. Reagent or Resourse

Antibodies	Cat No.	Company
H3K27me3 (C36B11) Rabbit mAb	9733S	Cell Signaling Technology
EZH2 XP (D2C9)	5246S	Cell Signaling Technology
SUZ12 (D-10)	SC-271325	Santa Cruz
EED	09-727	Millipore
PRKCBP1/ZMYND8	A302-089A	Bethyl Lab Inc.
Phospho-EZH2 (S21)	IHC-00388	Bethyl Lab Inc.
Phospho-EZH2 (Thr345)		Homemade
Phospho-EZH2 (Thr487)	PA5-105660	Invitrogen
ZMYND8	11633-1-AP	Proteintech
HIF1α	66730-1-Ig	Proteintech
HIF-2α (D9E3) Rabbit	7096S	Cell Signaling Technology
Histone H3	ab1791	abcam
ERK2 (D-2)	SC-1647	Santa Cruz
C-myc (9E10)	SC-40	Santa Cruz
FLAG M2 antibody	F-3165	Sigma
Phospho-Serine/Threonine mAb	612548	BD Biosciences
V5-Probe (C9)	SC-271944	Santa Cruz
CDC2 (POH1) (CDK1)	9116	Cell Signaling Technology
RB	554136	BD Biosciences
FOXM1(D12D5)	5436	Cell Signaling Technology
**Chemicals and Recombinant Proteins**	**Cat No.**	**Company**
Polyethylenimine (PEI)	Cat#408727	Sigma-Aldrich
Lipofectamine 2000 reagent	Cat#11668500	Thermo Fisher
Polybrene	Cat#TR-1003-G	Sigma-Aldrich
Cobalt(II) chloride hexahydrate	Cat#AC423570050	Fisher scientific
Roscovitine	Cat#557360	Sigma-Aldrich
RO-3306	Cat#sc-358700	Santa Cruz
**Critical Commercial Assays**	**Cat No.**	**Company**
KOD Plus Mutagenesis Kit	Cat#F0936K	Toyobo
Lambda Protein Phosphatase	Cat#P0753S	NEB
In vitro transcription/translation System	Cat#L1170	Promega

Table S3. Oligonucleotide sequences of qPCR primers

ZMYND8	Forward	GGGTTTATCACGCTAAGTGTCTG
	Reverse	GGCTTTACTCTGGGTCTCGATG
EZH2	Forward	CCCTGACCTCTGTCTTACTTGTGGA
	Reverse	ACGTCAGATGGTGCCAGCAATA
HOXA3	Forward	TGCAAAAAGCGACCTACTACGA
	Reverse	CGTCGGCGCCCAAAG
RUNX2	Forward	GTGCCTAGGCGCATTTCA
	Reverse	GCTCTTCTTACTGAGAGTGGAAGG
TCF7	Forward	CTTCGACCGCAACCTGAA
	Reverse	TTGATGGTTGGCTTCTTGG
MMP2	Forward	TGAGCTATGGACCTTGGGAGAA
	Reverse	CCATCGGCGTTCCCATAC
MMP7	Forward	GGGACATTCCTCTGATCCTAATGC
	Reverse	GAATTACTTCTCTTTCCATATAGTTTCTGAATGC
MMP19	Forward	CAGCCTCGTTGTGGCCTAGA
	Reverse	ACCAGCCTGCACCTCTTGGA
MMP24	Forward	TGGATCAGACAACGATCGAGTG
	Reverse	AGTCAGGGCATAGCGCTTGTTTC

Table S4. Oligonucleotide sequences of ChIP-qPCR primers

RUNX2	Forward	CGTAGTAGTACACAACGCCG
	Reverse	GTTTCGTGTCTGTCTTCCCC
MMP2	Forward	AACCAGTCTTGCCCAATTTC
	Reverse	CCTTCTGCAAATGTGTAAGCC
